# SASA-CLIP: Structure-Aware Alignment with a Gaussian Prior for Fine-Grained Video Action Recognition

**DOI:** 10.3390/s26154865

**Published:** 2026-08-02

**Authors:** Xiaowei Han, Wenbao Si, Honghui Zhang, Maolin Yang, Lin Ma, Yibo Feng

**Affiliations:** 1 School of Computer and Information Engineering, Harbin University of Commerce, Harbin 150028, China; siwenbao@hrbcu.edu.cn (W.S.); zhanghonghuiy2025@hrbcu.edu.cn (H.Z.); yangmaoliny2025@hrbcu.edu.cn (M.Y.); malin2025@hrbcu.edu.cn (L.M.); fengyibo@hrbcu.edu.cn (Y.F.); 2 Heilongjiang Provincial Key Laboratory of Electronic Commerce and Information Processing, Harbin 150028, China

**Keywords:** deep visual recognition, fine-grained action recognition, video understanding, vision–language models, CLIP, cross-modal alignment

## Abstract

Fine-grained video action recognition remains challenging because action categories often differ only in subtle inter-class variations and complex temporal dynamics. Recent Contrastive Language–Image Pre-training (CLIP)-based extensions perform well on general action recognition, but they typically rely on early global pooling of video features. Such coarse representations discard the fine temporal cues that distinguish subtle actions, causing a granularity mismatch in cross-modal alignment. To address this, we propose Structure-Aware Semantic-Adaptive (SASA)-CLIP, a framework for multi-granular cross-modal alignment. SASA-CLIP adopts a dual-branch design: a coarse-grained branch captures the global context, while a fine-grained branch matches descriptors against individual frames before aggregation, rather than pooling features early. To keep this alignment temporally coherent, we introduce a Gaussian prior as a temporal structural constraint, encoding the inductive bias of local temporal continuity into the attention matrix to guide an ordered alignment of key action segments along the temporal axis. On Kinetics-400 (ViT-B/32), SASA-CLIP reaches a Top-1 accuracy of 81.37%, improving over the X-CLIP baseline by 0.97%; on HMDB-51 and UCF-101 (ViT-B/16), it reaches 74.0% and 96.81%, improving by 3.25% and 2.61%, respectively. It also transfers to the zero-shot setting, improving over the baseline on HMDB-51 and UCF-101. These results show that combining multi-granular representations with a temporal structural prior benefits fine-grained recognition, suggesting that SASA-CLIP is a practical option for real-world visual sensing applications such as intelligent surveillance and wearable activity monitoring.

## 1. Introduction

Video action recognition is a fundamental problem in computer vision and a key component of many vision-based sensing systems, such as intelligent surveillance, human–robot interaction, and wearable activity monitoring [1]. As visual sensors become widely deployed in these settings, recognizing human actions reliably has become increasingly important, and growing attention is now turning to its more demanding setting: fine-grained recognition. Fine-grained video action recognition aims to distinguish action categories that share highly similar visual appearances and differ only in subtle temporal cues [2], such as “Swimming Backstroke” versus “Swimming Breast Stroke” and “Golf Chipping” versus “Golf Putting”. These action pairs typically share nearly identical background scenes, whereas their essential differences lie primarily in the trajectories of body movements and the temporal ordering of key frames. Distinguishing such pairs therefore requires the model to capture how the motion evolves over time, not just what appears in each frame.

In recent years, Contrastive Language–Image Pre-training (CLIP) [3] has constructed a rich joint vision–language embedding space by performing contrastive learning over massive image–text pairs collected from the Internet, enabling models to achieve open-vocabulary visual recognition through natural language prompts. As a result, CLIP generalizes well across downstream tasks such as image classification, object detection, and segmentation. Building upon this foundation, recent studies in video understanding have increasingly explored how to transfer CLIP’s pre-trained knowledge to action recognition. Methods such as ActionCLIP [4], X-CLIP [5], and ViFi-CLIP [6] treat videos as sequences of consecutive frames, extracting and aggregating frame-level visual features, and leverage the prior knowledge from large-scale vision–language pre-trained models to guide the learning of spatio-temporal video representations, with consistent gains on general action recognition benchmarks. A key advantage of this paradigm is open-vocabulary recognition: by grounding actions in language, these models can also recognize categories not seen during training.

**Figure 1:**
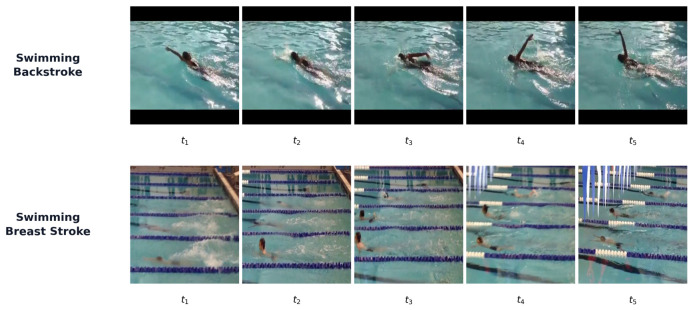
Granularity mismatch in X-CLIP: two visually similar swimming actions are cross-confused due to global alignment failing to capture frame-level temporal differences.

However, these methods have clear limitations in fine-grained settings. The core problem is the gap between relying on global alignment alone and the discrimination of fine-grained actions these tasks actually demand. This mismatch manifests itself in two main aspects:

The first is information loss from semantic compression. Most methods compress a whole video into a single global vector, using average pooling or a shallow Transformer, and then match it against a sentence-level text embedding. Such coarse aggregation smooths away the very frame-level variations that distinguish fine-grained actions. As illustrated in Figure 1, “Swimming Backstroke” and “Swimming Breast Stroke” share almost the same aquatic scene and differ only in how the arms move and at what rhythm. However, X-CLIP yields Softmax confidence scores below 0.45 for both classes (0.30 vs. 0.39 and 0.44 vs. 0.28, respectively), exhibiting pronounced discriminative uncertainty. This observation reveals the granularity mismatch that arises when fine-grained action recognition relies on global alignment alone.

Second, existing fine-grained alignment methods often suffer from computational redundancy and noise interference. Some methods attempt to introduce more fine-grained matching strategies; for instance, OST [7] adopts the Sinkhorn algorithm based on optimal transport to solve for the optimal matching between frames and descriptors. However, as noted in the original OST paper, the descriptors of a given action category are not always fully manifested in every video instance, and the descriptors generated by Large Language Models (LLMs) may contain hallucination-induced noise. Under such conditions, the forced global matching nature of Sinkhorn tends to introduce invalid alignments. Moreover, the Sinkhorn algorithm requires multiple iterations to converge, and its computational cost grows substantially as the sequence length increases [8]. To address these two limitations, we propose Structure-Aware Semantic-Adaptive (SASA)-CLIP, a structure-aware and semantically adaptive multi-granular video–language alignment framework. The core idea is to capture key action cues through fine-grained frame-level feature interaction and temporally structured alignment guided by semantic-adaptive priors.

The contribution of this work does not lie in separately introducing structured descriptors and a Gaussian prior into a CLIP-style alignment pipeline, but in the coupled design of the two. The centers of the prior are determined by the phase order of the descriptors, without any temporal annotation. In turn, the descriptors yield stable gains only under the temporal constraint provided by the prior. CLIP is pre-trained on static image–text pairs [3], and our empirical analysis shows that its embedding space is insensitive to the temporal order of the descriptors. We quantify the consequence of this property in frame-level descriptor matching: the peak matching positions of temporal descriptors from different phases almost coincide, and the matching degenerates into a temporally agnostic form (Section 3.3.2). The ablation results are consistent with this analysis. With the full descriptor set but no prior, Top-1 accuracy improves slightly while Top-5 accuracy falls below the baseline. Only after the prior is added do both metrics surpass the baseline (Section 4.2.3).

The main contributions of this work are summarized as follows:

- We propose SASA-CLIP, a model for fine-grained video–language alignment. A single global text representation cannot carry the full semantics of an action. To address this, we introduce temporal and spatial descriptors for each action category, which decompose the action into multi-granular semantic units for direct matching against frame-level visual features. This design avoids the loss of critical cues caused by early global pooling. The global matching in OST is driven by similarity alone, with no ordering constraint between frames and descriptors. We encode the intrinsic temporal structure of actions as an alignment constraint and inject it into the attention weights. This constraint takes the form of a closed-form bias and does not require iterative optimization.
- We design Gaussian-Prior Guided Attention (GPGA). It explicitly encodes the inductive bias of local temporal continuity into the cross-modal attention weights, guiding temporal descriptors toward an ordered alignment with their corresponding action phases. Prior work such as D3G [9] targets temporal localization tasks with annotations. The centers of the GPGA prior are directly determined by the phase order of the LLM-generated descriptors. Since the descriptors themselves carry the start, middle, and end phase order, the prior centers are obtained without any temporal annotation in the classification setting. The prior is not a hard constraint. A learnable coefficient α adjusts the relative weight between the prior term and the content similarity, so that the influence of the prior weakens when the temporal structure of a video deviates from the ideal phase order.
- Experiments on Kinetics-400, HMDB-51, and UCF-101 show that SASA-CLIP consistently outperforms the X-CLIP baseline, improving Top-1 accuracy by 0.97% on Kinetics-400 (ViT-B/32) and by 3.25% on HMDB-51 and 2.61% on UCF-101 (ViT-B/16); under zero-shot evaluation (ViT-B/16), it gains a further 4.3% on HMDB-51 and 3.1% on UCF-101.

## 2. Related Works

### 2.1. Video Action Recognition

Early work on video action recognition relied on deep convolutional networks to learn discriminative spatio-temporal representations. Methods such as C3D [10], I3D [11], and SlowFast [12] learn spatio-temporal feature representations through fully supervised training on large-scale datasets. Beyond deep networks, some methods address fine-grained action recognition with hand-crafted features and statistical models; for example, Yenduri et al. [13] use action-independent Gaussian mixture models with dynamic kernels to capture the subtle variations among fine-grained actions. Subsequently, Video Swin Transformer [14], ViViT [15], and TimeSformer [16] introduced the self-attention mechanism into the video domain, achieving notable progress in modeling long-range temporal dependencies. However, these methods all adopt a closed-set classification paradigm, where their generalization capability is inherently constrained by predefined category labels. To overcome this limitation, researchers have begun to leverage vision–language models (VLMs) for introducing semantic supervision signals, transforming video action recognition from a “label-mapping” task into a “video-text” semantic matching task.

Vision–language pre-trained models, represented by CLIP [3], perform contrastive learning over large-scale image–text pairs and have established a new paradigm for semantic-driven video action recognition. ActionCLIP [4] pioneered the “pre-train, prompt, and fine-tune” paradigm, reformulating action recognition as a video-text matching problem. X-CLIP [5] further proposed a cross-frame communication mechanism which explicitly introduces temporal modeling capability while preserving the pre-trained representation space of CLIP. ViFi-CLIP [6] demonstrated that full-parameter fine-tuning combined with a frame-averaging strategy can also establish a competitive baseline. Although these methods achieve strong performance on general datasets, they typically employ max pooling or average pooling to perform early-stage temporal compression, which may dilute critical local cues and limit the exploitation of frame-wise dynamic relationships and fine-grained motion patterns unique to videos. As illustrated in Figure 1 (introduced in Section 1), this global alignment paradigm exhibits inherent limitations when handling fine-grained actions, leading to insufficient discriminative capability in fine-grained scenarios.

### 2.2. Fine-Grained Cross-Modal Alignment for Action Recognition

To address the limitations of the global alignment paradigm, recent research has begun to explore fine-grained cross-modal alignment strategies. MP-Align [17] leverages an LLM to decompose complex activities into ordered step descriptions and achieves fine-grained alignment between video segments and texts through a multi-pathway alignment mechanism. RefAtomNet [18] addresses the referring action recognition task with a multi-stream architecture, introducing an agent attention mechanism for pixel-level grounding. OST [7] employs an LLM to generate spatial and temporal descriptors, and solves for the optimal matching between video frames and text descriptions using the Sinkhorn algorithm from optimal transport. BIKE [19] introduces bidirectional cross-modal knowledge into video representation learning through a knowledge distillation framework. FROSTER [20] further employs frozen CLIP as a powerful teacher model, preventing the fine-tuned feature space from excessively deviating from the pre-trained distribution, which effectively enhances generalization on open-vocabulary action recognition.

However, existing alignment strategies still face two main challenges. First, when computing frame-descriptor matching, these methods treat all frame positions as equivalent candidate alignment targets, without explicitly introducing any inductive bias regarding temporal structure. This means that a descriptor describing the start phase of an action may produce spuriously high matching scores with frames at the end of the video, while a descriptor for the end phase may erroneously attend to frames at the beginning. Such neglect of local temporal continuity weakens the model’s ability to separate critical action segments from redundant background frames during alignment. Second, as noted in the original OST [7] paper, the descriptors of a given action category are not always fully manifested in every video instance, and the descriptors generated by LLMs may contain hallucination-induced noise. Under such conditions, the forced global matching nature of Sinkhorn tends to introduce invalid alignments. Moreover, the Sinkhorn algorithm requires multiple iterations for convergence, and its computational cost grows substantially as the sequence length increases [8]. More recently, ActAlign [21] attempts to reformulate video classification as a sequence alignment problem using Dynamic Time Warping (DTW), achieving competitive results in the zero-shot setting. However, its performance heavily depends on the quality of LLM-generated sub-action scripts, and the rigid alignment constraint of DTW limits its adaptability to non-linear temporal variations. In addition, STDD [22] extends CLIP’s spatio-temporal understanding by constructing spatio-temporal text augmentation, but its focus lies in semantic enhancement on the text side, without explicitly introducing temporal structural constraints into the alignment mechanism itself.

Therefore, building fine-grained alignment that incorporates temporal structural awareness while maintaining low computational overhead remains an open problem.

### 2.3. Structural Priors for Temporal Modeling

As discussed in Section 2.2, existing fine-grained alignment methods have achieved notable progress at the semantic level; however, there remains substantial room for improvement in explicitly modeling temporal structures. Notably, the integration of structural priors into attention computation has been explored in several related fields, showing considerable potential for enhancing sequence and temporal modeling capabilities.

For positional bias in sequence modeling, Gaussian Transformer [23] introduces Gaussian distributions as priors into self-attention computation, thereby imposing locality constraints on attention weights and enabling the model to adaptively focus on specific local regions. ALiBi [24] replaces absolute positional encoding with a linear distance-decay bias, directly encoding the structural assumption that “the farther apart, the lower the attention” into the attention scores, thereby validating the general effectiveness of positional bias as an inductive bias. In the field of temporal action localization, ASL [25] introduces learnable Gaussian weights to model frame-level action sensitivity, enhancing attention to critical action frames while suppressing interference from background frames via Gaussian weighting. Along the temporal dimension of video understanding, D3G [9] incorporates a dynamic Gaussian prior to model the local temporal distribution of actions in temporal action localization, effectively mitigating the uncertainty of temporal boundaries. The aforementioned works demonstrate that temporal structural priors can provide significant benefits for action localization and sequence modeling. However, in temporal action localization, these priors rely on temporal annotations or inter-frame similarity to anchor their positions. Fine-grained video–language alignment for classification offers neither. In this work, the prior centers are instead derived from the phase order of the LLM-generated descriptors, which allows the Gaussian prior to serve as a structural constraint on cross-modal frame-descriptor alignment without any temporal supervision. The detailed design is presented in Section 3.

Table 1 summarizes representative methods along four dimensions: datasets, methodology, feature extraction, and performance evaluation. Early methods such as I3D and TimeSformer build on convolution or Transformer architectures and learn spatio-temporal representations under closed-set supervision, where recognition is limited to a predefined label set. CLIP-based methods introduce language supervision and enable the recognition of unseen categories. ActionCLIP and X-CLIP adapt CLIP through prompt learning and cross-frame modeling, while OST and BIKE improve the alignment through optimal transport and knowledge distillation, respectively. SASA-CLIP decomposes an action into multi-granular descriptors and aligns them with frame-level features under a Gaussian temporal prior, strengthening fine-grained temporal discrimination while remaining applicable to the zero-shot setting.

## 3. Methodology

This section elaborates on the proposed method in detail. We first provide a brief overview of the overall architecture in Section 3.1, including the coarse-grained branch (Section 3.1.1) and the fine-grained branch (Section 3.1.2). Section 3.2 and Section 3.3 then describe the construction of multi-granular semantic prototypes and the design of the cross-modal fine-grained alignment module, respectively. Section 3.4 introduces the adaptive gating fusion mechanism used for feature aggregation. Finally, the optimization objective and training strategy are presented in Section 3.5.

### 3.1. Overview of the Framework

**Figure 2:**
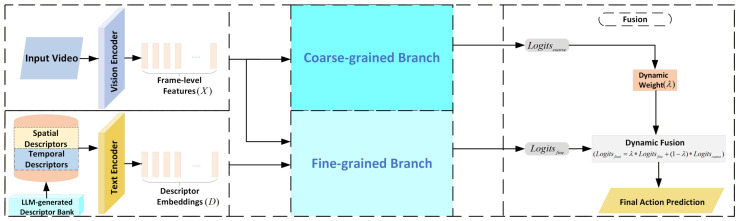
Overview of the SASA-CLIP architecture. A coarse-grained branch and a fine-grained branch are adaptively fused; their internal structures are detailed in the following figures.

We propose SASA-CLIP (Structure-Aware Semantic-Adaptive), illustrated in Figure 2. The core design principle is to elevate cross-modal alignment from the conventional “video-descriptor” level (where a globally pooled video feature is matched against a single text embedding) to the more fine-grained “frame-descriptor” level, while overcoming the unordered nature of traditional matching through a structural constraint based on a Gaussian prior. This design preserves the generalization capability inherited from CLIP while strengthening the model’s ability to capture subtle temporal action cues, thereby improving its performance on fine-grained action recognition.

As shown in Figure 2, SASA-CLIP adopts a dual-branch parallel cooperative architecture. During the feature extraction stage, the input video and the set of spatio-temporal descriptors generated by a large language model are processed by the vision encoder and the text encoder, respectively, producing frame-level visual feature sequences and a descriptor-embedding matrix. The two branches model video semantics from complementary perspectives and are adaptively fused at the output stage.

The two branches are designed to be complementary. The coarse-grained branch matches a global video representation against class-level text. It performs well on categories that are dominated by scene or global appearance, but cannot separate fine-grained actions whose differences lie only in subtle temporal cues, as shown in Figure 1. The fine-grained branch matches frame-level features against multi-granular descriptors and captures these local differences. When used in isolation, however, it lacks a stable global reference and is more sensitive to descriptor noise. Combining the two branches allows their global and local discrimination to compensate for each other. The outputs are merged by adaptive fusion according to the alignment confidence.

#### 3.1.1. Coarse-Grained Branch

**Figure 3:**
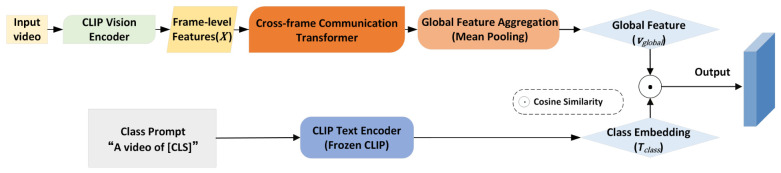
Illustration of the coarse-grained branch.

As shown in Figure 3, this branch follows the base architecture of X-CLIP [5] and aims to extract global discriminative features thereby providing a stable performance baseline for the overall framework. Specifically, the input video is uniformly sampled into a sequence of *T* frames, and the per-frame feature representations are extracted via a CLIP vision encoder initialized from pre-trained weights. Following the design of X-CLIP, a Cross-frame Communication Transformer is then introduced to model temporal dependencies while maintaining the consistency of the original CLIP pre-trained feature space. This module enables each frame to integrate the full-sequence context through fully connected self-attention interactions across frames, and the global video feature representation $v_{global}$ is finally obtained by mean pooling.

At the same time, the class label (e.g., “Archery”) is fed into the CLIP text encoder through a standard prompt template (“A video of [CLS]”) to obtain the class textual feature representation $t_{class}$. The matching score between the video feature and the text feature is then computed via cosine similarity, yielding the coarse-grained classification logits $Logits_{coarse}$. This branch generally maintains high recognition accuracy on categories dominated by scene or global appearance cues, thereby providing a reliable performance lower bound and reference baseline for subsequent fine-grained modeling.

#### 3.1.2. Fine-Grained Branch

**Figure 4:**
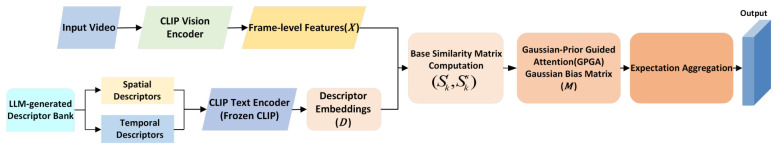
Illustration of the fine-grained branch.

As shown in Figure 4, this branch constitutes the core component of our method. Unlike the coarse-grained branch, which performs early aggregation of frame features, the fine-grained branch preserves the complete frame-level feature sequence and an extended semantic descriptor bank generated by an LLM. A Gaussian-Prior Guided Attention (GPGA) mechanism is then applied to explicitly encode the inductive bias of local temporal continuity into the attention weights. After expectation aggregation, this branch outputs the fine-grained classification logits $Logits_{fine}$. The detailed design of each submodule will be elaborated in Section 3.2 and Section 3.3.

### 3.2. Multi-Granularity Semantic Prototype Construction

**Figure 5:**
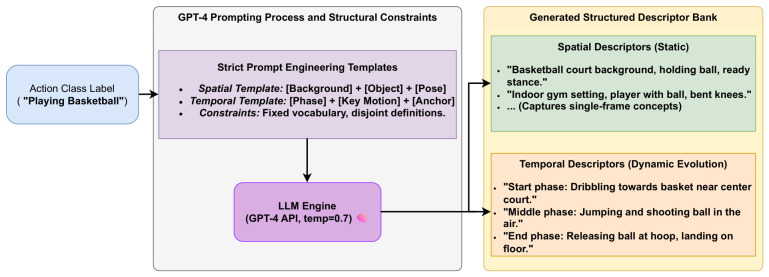
Construction pipeline of the multi-granularity semantic prototypes.

In video action recognition, relying solely on brief category names (e.g., “Playing Basketball”) for video-text alignment often leads to semantic insufficiency and ambiguity. To enhance the discriminability of the textual modality, we leverage a large language model (OpenAI GPT-4.1 API with temperature =0.7) [26] to generate a set of fine-grained semantic prototypes for each action category, which can be aligned with visual features. Figure 5 illustrates the construction pipeline of our descriptor bank and the structural constraints imposed on the spatio-temporal descriptors.

For each action category, $M_{s}$ spatial descriptors and $M_{t}$ temporal descriptors are generated through structured prompt templates ($M_{s}=M_{t}=3$). These descriptors are strictly organized along two orthogonal semantic dimensions, thereby enhancing semantic coverage while reducing inter-dimensional coupling. Furthermore, to prevent hallucinated outputs from the LLM that could cause the generated descriptors to become overly divergent, we design prompt templates with explicit vocabulary and syntactic constraints (Prompt Engineering), thereby constraining the output of the large language model at the semantic-structural level.

Spatial descriptors aim to capture static visual elements identifiable from a single frame without temporal context, such as scene backgrounds, objects/tools, human poses, or spatial relations. They provide frame-level visual anchors that enable the model to establish stable cross-modal correspondences at the local visual level. Their generation follows a fixed template as shown in Figure 5. Meanwhile, we impose semantic constraints to forbid the use of any temporal-order words or process-oriented connectives (e.g., “then”, “after”, “while”); verbs are restricted to stative or postural expressions (e.g., “holding”, “standing”, “bending”) to avoid implicit descriptions of cross-temporal changes. In addition, scene elements must be directly visible, and vague expressions such as “some” and “kind of” are prohibited. These constraints enable spatial descriptors to better exploit the representational capacity of the CLIP vision encoder for scenes, objects, and poses, thereby providing reliable contextual information for subsequent fine-grained alignment.

Temporal descriptors are designed to characterize the evolution of an action along the temporal axis, with particular emphasis on phase transitions and local motion patterns of the action, serving as the core semantic basis for temporal reasoning in the fine-grained branch. By explicitly encoding process information, temporal descriptors enable the model to distinguish between visually similar action categories that differ primarily in their temporal dynamics. As shown in Figure 5, the content of each temporal descriptor mainly consists of three components: *action phase*, *key motion*, and *spatial anchor*. Their generation is likewise constrained by a restricted vocabulary and structured expressions: the *action phase* must contain a phase indicator (e.g., “start”, “middle”, or “end”); the *key motion* must be a directly observable action phrase. In addition, each temporal descriptor must include an explicit spatial anchor (e.g., an object, body part, or scene reference) to ensure that the dynamic process remains alignable with static visual content.

Finally, for each action category, we extract textual features using a frozen CLIP text encoder to construct the descriptor embedding matrix. This matrix injects human prior knowledge into the model in a structured form, guiding the model to focus on more discriminative spatio-temporal details during alignment and providing a semantic foundation for cross-modal alignment and adaptive fusion.

During the encoding stage, we reformulate the text template into the form “A video of {Class Name} usually includes: {Action Descriptor}.” By explicitly introducing contextual information as a semantic anchor, this template projects homogeneous action semantics into more separable semantic subspaces, substantially reducing inter-class similarity and enabling the fine-grained branch to provide effective discriminative signals. Such a structured generation paradigm provides the model with richer semantic supervisory signals with explicit temporal logic. The complete prompt templates and the full set of spatial and temporal descriptors for all action categories are provided in the Supplementary Materials.

### 3.3. Structure-Aware Semantic-Adaptive Alignment Module

This module is the key component of our method, designed to establish fine-grained, structure-aware cross-modal correspondences between frame-level visual evidence and multi-descriptor semantic prototypes. We propose a SASA alignment mechanism to address the dilution of critical local action cues caused by coarse-grained temporal aggregation. While maintaining low computational complexity, the mechanism incorporates Gaussian-Prior Guided Attention (GPGA) to impose explicit constraints on the temporal structure of actions.

Unlike purely similarity-based matching approaches that align frames and descriptors based on appearance alone, our approach introduces a temporal inductive bias, enabling descriptors to focus on semantically appropriate and temporally coherent regions along the temporal axis. The module ultimately outputs fine-grained classification evidence that complements the coarse-grained branch (the fusion strategy is described in Section 3.4).

#### 3.3.1. Base Similarity Matrix Computation

Given an input video, the frame-level feature sequence extracted by the visual encoder is defined as in Equation (1):

(1) $$X=\left\{x_{1},x_{2},\ldots ,x_{T}\right\}\in R^{T\times D}$$

where *T* denotes the number of sampled frames and *D* is the feature dimension. For a given action category *k*, the multi-granularity semantic prototype library constructed in Section 3.2 contains a set of temporal descriptors and a set of spatial descriptors, as defined in Equation (2):

(2) $$D_{k}^{t}=\left\{d_{k,1}^{t},\ldots ,d_{k,M_{t}}^{t}\right\},\;D_{k}^{s}=\left\{d_{k,1}^{s},\ldots ,d_{k,M_{s}}^{s}\right\}$$

where $M_{t}$ and $M_{s}$ denote the numbers of temporal and spatial descriptors, respectively, and both $d_{k,j}^{t}$ and $d_{k,j}^{s}$ are obtained from the frozen CLIP text encoder.

To eliminate differences in vector magnitude and to remain consistent with the cosine similarity measure of the CLIP pre-trained space, we first normalize the visual features and the two types of textual descriptors, as defined in Equation (3):

(3) $${\hat{x}}_{i}=\frac{x_{i}}{∥x_{i}{∥}_{2}},\;{\hat{d}}_{k,j}^{t}=\frac{d_{k,j}^{t}}{∥d_{k,j}^{t}{∥}_{2}},\;{\hat{d}}_{k,j}^{s}=\frac{d_{k,j}^{s}}{∥d_{k,j}^{s}{∥}_{2}}$$

Based on the normalized features, the dot-product similarity between the frame sequence and the two types of descriptors is computed, yielding two base similarity matrices, as defined in Equations (4) and (5):

(4) $$S_{k}^{t}\left[i,j\right]={\hat{x}}_{i}^{⊤}{\hat{d}}_{k,j}^{t},\;i\in \left\{1,\ldots ,T\right\},\;j\in \left\{1,\ldots ,M_{t}\right\}$$

(5) $$S_{k}^{s}\left[i,j\right]={\hat{x}}_{i}^{⊤}{\hat{d}}_{k,j}^{s},\;i\in \left\{1,\ldots ,T\right\},\;j\in \left\{1,\ldots ,M_{s}\right\}$$

where $S_{k}^{t}\in R^{T\times M_{t}}$ and $S_{k}^{s}\in R^{T\times M_{s}}$. Each matrix element [i,j] characterizes the matching strength between the *i*-th frame and the *j*-th semantic descriptor in the shared embedding space. However, $S_{k}^{t}$ and $S_{k}^{s}$ are still essentially content-level matching results. As analyzed in Section 3.3.2, in the CLIP pre-trained embedding space, the matching patterns between temporal descriptors of different phases and video frames are nearly identical.

#### 3.3.2. Gaussian-Prior Guided Attention (GPGA)

To quantify the temporal agnostic behavior of $S_{k}^{t}$, we conducted an empirical analysis on the Kinetics-400 validation set using the CLIP model. For each video, we computed the cosine similarity between all frames and the three temporal descriptors of the corresponding action category (which correspond to the *start*, *middle*, and *end* phases of the action, respectively), and recorded the normalized position on the time axis [0,1] of the frame that achieves the maximum similarity with each descriptor. We denote this normalized peak position as μ for each descriptor.

The results are presented in Table 2. The expected μ denotes the ideal positions (0.00, 0.50, 1.00) under temporally aware matching, while the empirical μ is the mean peak position observed across the validation set. As shown, the peak positions of the three descriptors largely overlap with each other, deviating significantly from the ordered distribution, and cannot be effectively fitted by a Gaussian function. This observation reveals a critical issue: since CLIP is pre-trained on static image–text pairs, its embedding space lacks awareness of the temporal evolution of action sequences, and descriptors corresponding to different phases exhibit highly consistent feature directions. As a result, CLIP cannot exploit the phase order encoded in the descriptors, causing the frame-descriptor matching to degenerate into temporally agnostic matching. If alignment is performed directly under such conditions, the visual encoder tends to collapse all frame features toward a single descriptor that bears the highest similarity to the static scene, thereby weakening its discriminability with respect to the underlying action-phase structure. Therefore, introducing an explicit temporal structural prior is necessary. This observation is further validated in Section 4.2 of the experiments.

Inspired by D3G [9], which employs dynamic Gaussian priors for temporal calibration in temporal sentence grounding, we introduce a similar structured inductive bias into the cross-modal alignment of fine-grained action recognition. The core assumption is that temporal descriptors corresponding to the same action phase should exhibit local continuity and a relatively stable distribution trend along the temporal axis. This assumption originates from the intrinsic temporal structure of human actions, which generally follow an ordered phase pattern of preparation, execution, and completion [27]. The key difference from D3G lies in the task setting: D3G targets temporal localization tasks with single-frame annotations and dynamically adjusts the Gaussian center by exploiting inter-frame feature similarity, whereas our work addresses action classification without temporal annotations, where the phase order of temporal descriptors is determined by the semantic sequence generated by the LLM. Therefore, we adopt deterministic descriptor-index-based centers together with a shared global standard deviation, which injects temporal-ordering constraints while maintaining low computational overhead.

Specifically, we predefine an ideal normalized temporal center $\mu_{j}$ for each temporal descriptor, whose value is determined by the descriptor’s relative position within the phase sequence, as defined in Equation (6):

(6) $$\mu_{j}=\frac{j-1}{M_{t}-1},\;j\in \left\{1,2,\ldots ,M_{t}\right\}$$

For a video frame sequence, we normalize the frame index *i* as $\bar{i}=\left(i-1\right)/\left(T-1\right)$. Based on this, we construct a Gaussian bias matrix, as defined in Equation (7):

(7) $$M\left[i,j\right]=-\frac{{\left(\bar{i}-\mu_{j}\right)}^{2}}{2\sigma^{2}}$$

where σ is the standard deviation of the Gaussian distribution, controlling the width of the temporal window. A smaller σ produces sharper phase constraints, while a larger σ allows a certain degree of temporal drift to accommodate variations in action speed (e.g., differences between fast and slow actions).

We then inject this structural prior into the similarity logits to form the corrected alignment scores, as defined in Equation (8):

(8) $${\tilde{S}}_{k}^{t}\left[i,j\right]=S_{k}^{t}\left[i,j\right]+\alpha \cdot M\left[i,j\right]$$

where α is a learnable parameter that adaptively balances content matching and the temporal prior. This formulation can be interpreted from a Bayesian inference perspective: the first term $S_{k}^{t}\left[i,j\right]$ corresponds to the degree of support from visual evidence, while the second term α·M[i,j] corresponds to the structural preference for the temporal ordering of descriptors, thereby strengthening the temporal consistency of alignment without significantly increasing computational cost.

It is also worth noting that the Gaussian prior is applied only to temporal descriptors and not to spatial descriptors. This decoupled design originates from the fact that spatial descriptors characterize static intra-frame cues that lack temporal ordering, and imposing temporal constraints on them would introduce inappropriate restrictions.

#### 3.3.3. Expectation Aggregation

Based on the corrected temporal similarity matrix ${\tilde{S}}_{k}^{t}$ obtained from GPGA and the spatial similarity matrix $S_{k}^{s}$, expectation aggregation is employed to integrate frame-level features into classification scores. Specifically, softmax normalization is applied along the descriptor dimension to obtain the frame-descriptor matching weights, as defined in Equations (9) and (10):

(9) $$A_{k}^{t}\left[i,j\right]=\frac{\exp({\tilde{S}}_{k}^{t}\left[i,j\right]/\tau_{att})}{\sum_{j^{\prime }=1}^{M_{t}}\exp\left({\tilde{S}}_{k}^{t}\left[i,j^{\prime }\right]/\tau_{att}\right)}$$

(10) $$A_{k}^{s}\left[i,j\right]=\frac{\exp(S_{k}^{s}\left[i,j\right]/\tau_{att})}{\sum_{j^{\prime }=1}^{M_{s}}\exp\left(S_{k}^{s}\left[i,j^{\prime }\right]/\tau_{att}\right)}$$

where $\tau_{att}$ is the temperature parameter. The fine-grained classification score for category *k* is finally obtained by expectation aggregation over all descriptors, as defined in Equation (11):

(11) $$z_{k}^{fine}=\frac{1}{M_{t}}\sum_{j=1}^{M_{t}}\sum_{i=1}^{T}A_{k}^{t}\left[i,j\right]\cdot {\tilde{S}}_{k}^{t}\left[i,j\right]+\frac{1}{M_{s}}\sum_{j=1}^{M_{s}}\sum_{i=1}^{T}A_{k}^{s}\left[i,j\right]\cdot S_{k}^{s}\left[i,j\right]$$

This aggregation strategy ensures that only frames falling within reasonable temporal windows and relevant to critical semantic descriptors contribute more substantially to the classification, while maintaining computational efficiency, thereby improving fine-grained action discrimination. As shown by the visualization analysis in Section 4.2.3, after end-to-end training under the GPGA constraint, the matching matrix $A_{k}^{t}$ exhibits a pronounced diagonal trend: start-phase descriptors achieve the highest activation in the early portion of the video, middle-phase descriptors in the middle portion, and end-phase descriptors in the latter portion. This sharply contrasts the pre-training state and shows what GPGA contributes. With the right inductive bias in place, the visual encoder learns temporally discriminative representations that standard global alignment, on its own, rarely produces.

### 3.4. Adaptive Fusion

**Figure 6:**
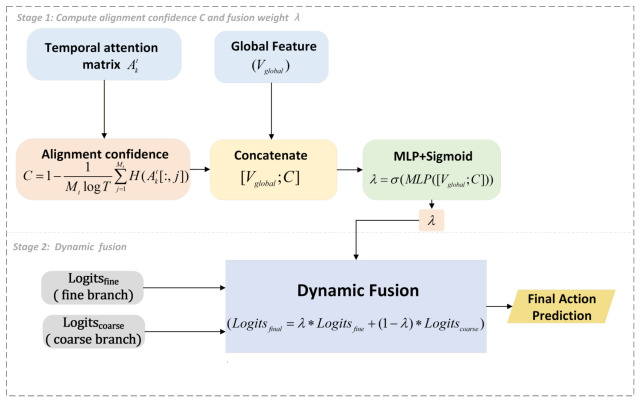
Illustration of the adaptive fusion mechanism.

After obtaining the coarse-grained logits $Logits_{coarse}$ and the fine-grained logits $Logits_{fine}$, effectively integrating the complementary information from the two branches becomes crucial for the overall recognition performance. Fixed-ratio weighted fusion is a simpler approach, but it implicitly assumes that all categories rely equally on fine-grained cues, ignoring inter-class differences. For action categories that are strongly scene-dependent, global semantic and background cues alone are sufficient for accurate recognition, and over-introducing fine-grained matching may instead amplify noise and lead to performance degradation. To address this, we design an adaptive fusion mechanism that dynamically adjusts the fusion weights between the coarse-grained and fine-grained logits according to the alignment confidence estimated by the GPGA module, as illustrated in Figure 6.

Whether the fine-grained branch can effectively distinguish between highly similar actions largely depends on the quality of the alignment between temporal descriptors and video frames. Therefore, we use the concentration of the GPGA attention matrix $A_{k}^{t}$ as a measure of alignment quality. Specifically, the alignment degree *C* is defined as in Equation (12):

(12) $$C=1-\frac{1}{M_{t}\log T}\sum_{j=1}^{M_{t}}H\left(A_{k}^{t}\left[:,j\right]\right)$$

where H(·) denotes the entropy function, $A_{k}^{t}\left[:,j\right]$ represents the attention distribution of the *j*-th temporal descriptor over *T* video frames, and $M_{t}$ is the number of temporal descriptors. When the distribution tends toward uniform, a temporal descriptor matches all frames almost equally, and *C* approaches 0, indicating that the fine-grained branch fails to establish a discriminative and temporally localized alignment. Conversely, when the distribution is highly concentrated within a specific temporal window, *C* approaches 1, indicating that the temporal descriptor has successfully matched the corresponding action phase, and the fine-grained branch is assigned a higher fusion weight.

Subsequently, the global visual feature $v_{global}$ extracted from the coarse-grained branch is concatenated with the alignment signal *C*, and the resulting vector is fed into a multi-layer perceptron (MLP) network [28,29] to produce the sample-specific dynamic fusion parameter λ. Finally, the video action prediction logits are obtained by adaptively combining the outputs of the coarse-grained and fine-grained branches in a sample-dependent manner, as defined in Equations (13) and (14):

(13) $$\lambda =\sigma (\mathrm{MLP}\left(\left[v_{global};C\right]\right))$$

(14) $$Logits_{final}=\lambda \cdot Logits_{fine}+\left(1-\lambda \right)\cdot Logits_{coarse}$$

### 3.5. Optimization Objective and Training Strategy

The model is trained end-to-end with a joint optimization strategy. The loss function adopts the standard symmetric cross-entropy loss, which is commonly used in CLIP-based models for contrastive learning. Given a batch of *N* videos and their corresponding *N* class labels (along with the generated descriptor sets), we compute the bidirectional contrastive losses (video-to-text and text-to-video) and sum them as the final training objective, as defined in Equations (15) and (16):

(15) $$L=\frac{1}{2}\left(L_{v2t}+L_{t2v}\right)$$

(16) $$L_{v2t}=-\frac{1}{N}\sum_{i=1}^{N}\log\frac{\exp(Logits_{final}\left(i,y_{i}\right)/\tau_{con})}{\sum_{j=1}^{N}\exp\left(Logits_{final}\left(i,y_{j}\right)/\tau_{con}\right)}$$

where $\tau_{con}$ is the temperature parameter of contrastive learning, used to adjust the smoothness of the similarity distribution. Through this design, the fine-grained cross-modal alignment objective directly drives the visual encoder to learn frame-level representations with greater semantic discriminability, forming a closed-loop optimization with the high-level semantic constraints and thereby achieving a synergistic improvement of feature learning and semantic alignment.

## 4. Experiments

In this section, we conduct experiments on three widely used video action recognition datasets, namely HMDB-51, UCF-101, and Kinetics-400, to comprehensively evaluate the effectiveness and generalization capability of the proposed SASA-CLIP model. We first describe the experimental setup in Section 4.1, including dataset details, implementation configurations, and evaluation protocols. We then present detailed ablation studies on the key components of our model, namely the dual-branch collaboration and the GPGA module, in Section 4.2. Section 4.3 presents the main results and comparisons with state-of-the-art methods. Extensive experiments demonstrate that SASA-CLIP achieves competitive Top-1 accuracy on all three benchmark datasets, consistently outperforming the X-CLIP baseline and obtaining notable improvements on the fine-grained action categories of HMDB-51.

### 4.1. Experimental Setup

#### 4.1.1. Datasets

To comprehensively evaluate the effectiveness of SASA-CLIP, we conduct experiments on three widely used video action recognition benchmarks, as summarized in Table 3:

**HMDB-51** [30]: This dataset contains 6849 web-sourced video clips spread across 51 action categories. The clips center on facial actions, body movements, and human-object interactions, run at 320 × 240 resolution, and last about 5 s on average. With more than one hundred clips per category, it is a good testbed for fine-grained recognition when training data are scarce.

**UCF-101** [31]: A small-to-medium-scale dataset containing 101 action categories, primarily used to evaluate the model’s transfer capability in few-shot and zero-shot scenarios.

**Kinetics-400** [11]: A large-scale dataset consisting of approximately 240k training videos and 20k validation videos, covering 400 human action categories. Each video lasts approximately 10 s. Due to its large scale and category diversity, Kinetics-400 serves as an important benchmark for evaluating the robustness and scalability of video action recognition methods.

#### 4.1.2. Implementation Details

To ensure fair comparison with the X-CLIP baseline, we adopt the CLIP pre-trained vision encoder as the backbone in all experiments. The model is trained on four NVIDIA RTX 4090D GPUs. The video input is uniformly sampled into *T* frames, with T∈{8,16,32} depending on the experimental setting, and each frame is resized to 224×224 pixels. Specifically, we use ViT-B/32 and ViT-B/16 backbones for the Kinetics-400 supervised experiments, ViT-B/16 for the HMDB-51 and UCF-101 supervised experiments, and ViT-B/16 with T=32 for the zero-shot experiments.

Regarding the training strategy, the CLIP text encoder is kept frozen to preserve the linguistic prior knowledge learned during large-scale vision–language pre-training, while the CLIP vision encoder is fully fine-tuned together with the cross-frame communication Transformer and the SASA module to adapt to the visual representation learning required for video tasks. To accommodate the differing learning dynamics of pre-trained and newly introduced parameters, we adopt a layer-wise learning rate strategy in which the newly introduced modules (cross-frame communication Transformer and SASA module) use a 10× learning rate multiplier relative to the CLIP backbone. The detailed training hyperparameters are summarized in Table 4.

On the textual side, we leverage a large language model (GPT-4.1) to generate M=6 hierarchical descriptors for each action category, consisting of three spatial descriptors and three temporal descriptors. The construction of the descriptor bank is subject to strict syntactic and semantic constraints, ensuring that the generated descriptors are visually observable, action-relevant, and phase-aware. During training, the total loss function consists of bidirectional contrastive losses between video-to-text and text-to-video directions, optimized via symmetric cross-entropy. For inference, we adopt the multi-view evaluation protocol with 4 temporal clips and 3 spatial crops.

### 4.2. Ablation Experiments and Analysis

To quantitatively validate the effectiveness of the core design components of SASA-CLIP, namely the dual-branch collaboration, temporal structural awareness, and multi-granularity semantic prototypes, this section conducts ablation studies under a unified setting. All models in this section adopt ViT-B/32 and ViT-B/16 as the backbone architectures and are trained on four NVIDIA RTX 4090D GPUs. We follow the single-view evaluation protocol to ensure a fair comparison while optimizing computational resource utilization.

#### 4.2.1. Fine-Grained Branch Ablation and Analysis

The core of our method lies in the complementary collaboration between the coarse-grained and fine-grained branches: the coarse-grained branch captures global contextual information, while the fine-grained branch captures local temporal dynamics through structure-aware descriptor alignment. To validate the effectiveness of the fine-grained branch, we compare the coarse-grained-only baseline (i.e., X-CLIP) with the complete dual-branch fusion model (SASA-CLIP) on the Kinetics-400 dataset. Both configurations adopt ViT-B/32 and ViT-B/16 as the visual backbone, with the number of input frames set to 8 and 16, respectively.

As shown in Table 5, the introduction of the fine-grained branch yields consistent performance improvements across all evaluated configurations. Specifically, SASA-CLIP improves the Top-1 accuracy by 0.97% (80.40% → 81.37%) under the ViT-B/32, T=8 setting and by 0.90% (84.70% → 85.60%) under the ViT-B/16, T=16 setting. Moderate but consistent gains are also observed under the intermediate configurations: 0.60% improvement under ViT-B/32, T=16 (81.10% → 81.70%) and 0.43% improvement under ViT-B/16, T=8 (83.80% → 84.23%). Top-5 accuracy follows the same trend, with the largest gain of 0.24% observed under the ViT-B/16, T=16 setting (96.80% → 97.04%). These results indicate that the fine-grained branch provides stable and complementary benefits to the coarse-grained branch across different backbone capacities and temporal sampling rates, demonstrating its effectiveness in enhancing fine-grained video action recognition.

The above experiments verify the overall gain of the fine-grained branch under different configurations. To further quantify the individual contributions of the coarse-grained and fine-grained branches, we evaluate a single trained model under the ViT-B/32, T=8 setting, using only the coarse-grained output (λ=0), only the fine-grained output (λ=1), and the full fusion, respectively, as shown in Table 6. With the coarse-grained output only (λ=0), the Top-1 accuracy is 80.53%, slightly higher than the independently trained X-CLIP baseline (80.40%). Both configurations share essentially the same structure. The difference is that the visual encoder of the full model also receives gradients from the fine-grained branch during end-to-end training, so that the learned frame-level representations are also beneficial to the global discrimination of the coarse-grained branch. With the fine-grained output only (λ=1), the Top-1 accuracy drops to 79.82%, below the baseline. The fine-grained branch captures local temporal differences through frame-level descriptor matching, but lacks the global context provided by the coarse-grained branch. Some Kinetics-400 categories can be distinguished from scene and global semantic cues alone. For these categories, the fine-grained branch alone does not provide sufficient discriminative evidence. This shows that the fine-grained branch provides complementary local temporal discriminative information for the global representation, while its performance when used alone remains inferior to the full model. By combining the two branches through adaptive fusion, the full model obtains the best result of 81.37%, higher than any single-branch configuration, which verifies the effectiveness of the dual-branch cooperative design.

**Figure 7:**
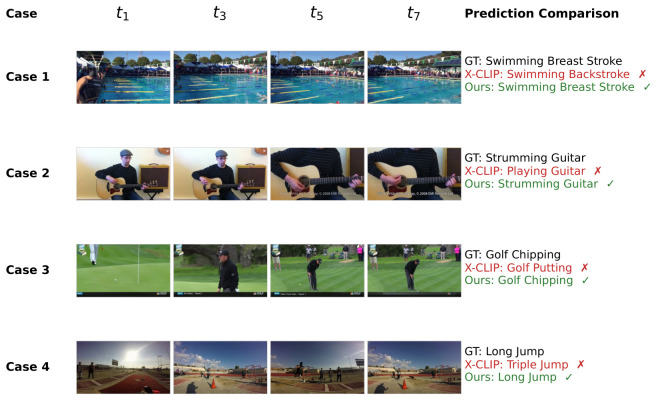
Qualitative examples where X-CLIP misclassifies but SASA-CLIP correctly identifies fine-grained actions across four confusion types: motion pattern, action granularity, action amplitude, and temporal structure. ✓ denotes a correct prediction and × denotes an incorrect prediction.

Figure 7 presents four representative cases where X-CLIP makes incorrect predictions while SASA-CLIP predicts correctly. *Swimming Breast Stroke* and *Swimming Backstroke* share an identical aquatic scene and differ only in swimming posture; X-CLIP misclassifies breaststroke as backstroke, while SASA-CLIP successfully distinguishes the two through temporal descriptor matching. Both *Strumming Guitar* and *Playing Guitar* involve a guitar as the central interaction object. SASA-CLIP’s spatial and temporal descriptors cooperatively capture the rhythmic reciprocating motion unique to strumming. *Golf Chipping* and *Golf Putting* exhibit nearly identical scenes and initial postures, and SASA-CLIP, by constraining the temporal descriptors within their corresponding temporal windows via the GPGA module, effectively captures the difference in swing amplitude. The core distinction between *Long Jump* and *Triple Jump* lies in the number of action phases: a single takeoff versus three consecutive jumps; this difference cannot be captured by single-frame features, and SASA-CLIP successfully recognizes the single-phase structure of a long jump by aligning temporal descriptors with the frame sequence in an ordered and structure-aware manner through GPGA.

The above experimental results and qualitative cases demonstrate that relying solely on global feature matching is insufficient to fully distinguish action categories with subtle dynamic differences. When facing highly similar actions, as illustrated in Figure 7, the global pooling used by the coarse-grained branch tends to dilute critical local cues, leading to reduced discriminative capability for visually similar action categories. Our method addresses this issue by introducing the fine-grained branch, which enhances the model’s spatio-temporal modeling capability from the textual side.

By constructing structured spatio-temporal semantic prototypes on the textual side, our method expands a single category label into multi-granular supervisory signals that explicitly encode temporal phases and visual anchors. Under the guidance of rich semantic priors and the temporal constraint of GPGA, the visual encoder is driven to learn to discriminate between highly similar actions. Building on the coarse-grained branch, the fine-grained branch supplements the critical local discriminative information needed for distinguishing easily confused actions, forming an effective complementarity with the coarse-grained branch.

To complement the successful cases above, we also examine the samples that SASA-CLIP still misclassifies. One source of the residual errors is the mismatch between the LLM-generated descriptors and individual instances. As noted in the original OST [7] work, the descriptors of an action category are not always fully manifested in every instance, and LLM-generated descriptors may contain hallucinated content. When matched at the frame level, such descriptors can inject misleading evidence into the fine-grained branch. We further examine how many such errors occur. On the Kinetics-400 validation set, only 9 samples are correctly classified by the coarse-grained branch but misclassified after fusion, indicating that the adaptive fusion suppresses descriptor-induced errors to a negligible level. Filtering low-quality descriptors by matching score, as discussed in Section 5, is a direct direction for further reducing these residual errors.

#### 4.2.2. Ablation on Descriptor Types and Fusion Strategy

The ablation in this section consists of two parts. The first part evaluates the individual contributions of the two descriptor types, spatial and temporal descriptors. The second part evaluates the fusion strategy between the coarse-grained and fine-grained branches, comparing fixed fusion with adaptive fusion. We conduct an ablation study on Kinetics-400 (ViT-B/32, T=8), with results shown in Table 7. To keep the descriptor type as the only variable, both single-descriptor configurations adopt a fixed branch fusion weight, and GPGA is applied only to temporal descriptors.

Using spatial descriptors alone, the Top-1 accuracy is 80.24%, slightly below the X-CLIP baseline (80.40%). Spatial descriptors characterize static information such as scene, objects, and poses, which is already covered by the global representation of the coarse-grained branch. As a result, introducing them alone brings little gain, while their frame-level matching adds extra noise. Using temporal descriptors alone, the Top-1 accuracy reaches 80.71%, above the baseline. Temporal descriptors encode the phase evolution of an action. Such fine-grained temporal cues are weakened when the coarse-grained branch aggregates frame features into a global representation. They remain essential for distinguishing fine-grained actions. This observation is consistent with OST [7], which likewise finds that temporal descriptors contribute more significantly than spatial ones. Under the fixed fusion strategy, combining the two descriptor types raises the accuracy to 81.06%, higher than either type alone, which indicates that the two are complementary.

The last two rows of Table 7 evaluate the fusion strategy between the coarse-grained and fine-grained branches, comparing fixed fusion with adaptive fusion. Under the full descriptor configuration, adaptive fusion reaches 81.37% Top-1 and 95.35% Top-5, higher than the 81.06% and 95.23% of fixed fusion. Fixed-ratio fusion assigns the same weight to the two branches across all categories. In contrast, adaptive fusion adjusts the weight between the coarse-grained and fine-grained branches per sample according to the alignment confidence from GPGA and therefore yields better results.

We further analyze the effect of the number of spatial descriptors on performance. It should be noted that the number of temporal descriptors is fixed at 3, corresponding to the start, middle, and end phases of an action, and is bound to the Gaussian prior centers of GPGA (Equation (6)). It is therefore not treated as an independent variable in this analysis. In contrast, spatial descriptors are mutually independent static visual dimensions, and their number can be examined separately. The results are shown in Table 8. When the number of spatial descriptors increases from 1 to 3, the Top-1 accuracy improves from 81.14% to 81.37%. This improvement is consistent with the design intent of spatial descriptors. A single descriptor is insufficient to cover multiple static visual dimensions such as scene, objects, and posture, whereas a moderate number of descriptors provides more comprehensive visual anchors. However, when the number increases to 5 and 7, the accuracy drops to 81.21% and 81.07%, respectively. This observation is consistent with the finding in OST [7] that an excessive number of descriptors aggravates the hallucination problem of large language models and introduces noisy descriptors. The reason is that the static semantic dimensions of a single action category are limited. When the number of descriptors exceeds the visual concepts that can be covered, the large language model generates repetitive or fabricated content. Despite these effects, the overall accuracy fluctuation remains within a narrow range (81.07% to 81.37%). This stability has two sources. First, the structured prompt template in Section 3.2 imposes vocabulary and syntactic constraints on descriptor generation, which limits the deviation of descriptor content. Second, as shown in Table 7, spatial descriptors mainly serve as a complement to the global representation. Considering both performance and noise, we set $M_{s}=3$.

Regarding the three-phase temporal design described above, its applicability under different video structures requires further clarification. The Gaussian prior assumes that action phases are temporally ordered along the time axis. This assumption is reasonable for most complete actions, but it does not always hold in cases such as trimmed clips, repetitive actions, shot changes, or actions where key evidence appears only briefly. Our method handles these cases through the following designs.

The Gaussian prior in Equation (8) is not imposed on the alignment process as a hard constraint. Instead, it is combined with content similarity through the learnable fusion coefficient α. When the temporal structure of a video deviates from the ideal phase order, α adaptively decreases during training, and the model relies more on the content matching between frames and descriptors than on the fixed temporal assumption. The Gaussian distribution adopted by the prior is itself a smooth soft window. Its standard deviation σ allows action phases to shift along the time axis, which accommodates differences in action tempo and starting position. At the branch fusion level, the adaptive fusion in Section 3.4 dynamically adjusts the weights according to the temporal alignment quality of each sample. When the temporal alignment quality of a video is low, the overall weight of the fine-grained branch decreases accordingly, and the inappropriate prior it may introduce is suppressed as well. Therefore, although the fixed phase assumption still has limitations on extremely irregular videos, the above mechanisms keep the three-phase temporal modeling robust under common conditions.

#### 4.2.3. Ablation Study and Analysis of the GPGA Module

The GPGA module is designed to address the unordered frame-descriptor matching problem in fine-grained alignment by injecting an inductive bias of local temporal continuity. To quantitatively validate the effectiveness of the GPGA module, we design the controlled experiments shown in Table 9, starting from the X-CLIP baseline and progressively introducing the fine-grained branch and the GPGA module to systematically analyze the contribution of each component.

To analyze the contribution of each component in detail, we conduct controlled experiments using ViT-B/32 as the backbone under two input configurations (T=8 and T=16). Starting from the X-CLIP baseline, we first introduce the fine-grained branch (i.e., SASA-CLIP w/o GPGA). Under the T=8 configuration, the Top-1 accuracy improves from 80.40% to 80.81% (+0.41%), while the Top-5 accuracy unexpectedly drops from 95.00% to 94.67% (−0.33%); the same pattern is observed under the T=16 configuration, where the Top-1 accuracy improves from 81.10% to 81.23% (+0.13%) but the Top-5 accuracy drops from 95.50% to 95.10% (−0.40%). This contrast between the Top-1 improvement and the Top-5 decline corroborates the empirical analysis in Section 3.3.2 (Table 2): without temporal structural priors, the matching between temporal descriptors and video frames is temporal agnostic. Although the fine-grained branch enhances the model’s discriminability over the most matched category (Top-1), the alignment noise introduced by unordered frame-descriptor matching impairs semantic recall across the top-ranked candidates, leading to a decline in Top-5 accuracy.

**Figure 8:**
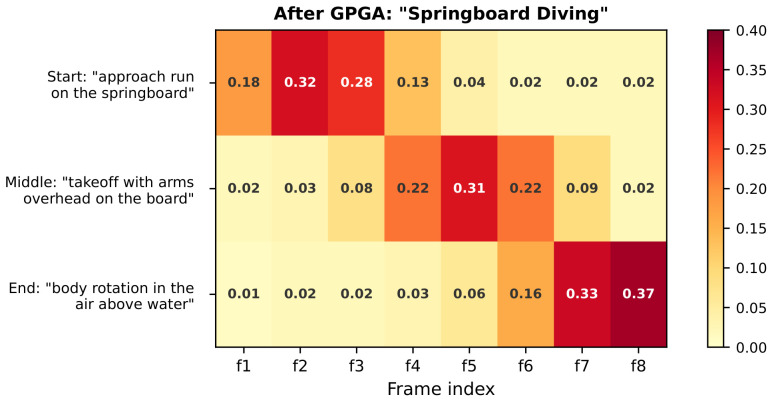
Temporal descriptor-frame matching distribution after applying the GPGA module on the action category *Springboard Diving*.

After further incorporating the GPGA module (i.e., the complete SASA-CLIP), the performance pattern changes significantly. Under the T=8 configuration, the Top-1 accuracy improves from 80.81% to 81.37% (+0.56%), and the Top-5 accuracy recovers from 94.67% to 95.35% (+0.68%); under the T=16 configuration, the Top-1 accuracy improves from 81.23% to 81.70% (+0.47%), and the Top-5 accuracy recovers from 95.10% to 95.67% (+0.57%). By explicitly injecting the inductive bias of local temporal continuity into the attention weights, the GPGA module constrains the start, middle, and end phase descriptors to focus on their corresponding temporal windows along the video (detailed visualization in Figure 8). This temporally ordered constraint effectively alleviates the unordered-collapse problem in fine-grained frame-descriptor alignment.

Figure 8 illustrates the working mechanism of the GPGA module. After incorporating GPGA, the inductive bias of local temporal continuity is explicitly encoded into the matching process. Taking *Springboard Diving* as an example, the matching matrix after the GPGA constraint exhibits a clear diagonal trend: the start-phase descriptor “approach run on the springboard” attends primarily to the early frames ($f_{1}$–$f_{3}$), the middle-phase descriptor “takeoff with arms overhead on the board” attends to the middle frames ($f_{4}$–$f_{6}$), and the end-phase descriptor “body rotation in the air above water” attends to the latter frames ($f_{7}$–$f_{8}$). Such ordered alignment enables the model to distribute attention more appropriately along the temporal axis, preventing fine-grained features from collapsing toward a single dominant semantic node.

More notably, the performance gain brought by GPGA (+0.56%/+0.47%) substantially exceeds that of the fine-grained branch alone (+0.41%/+0.13%), and only after introducing GPGA does the Top-5 accuracy surpass the baseline under both input configurations. This demonstrates that the GPGA module is not merely an auxiliary refinement to the fine-grained branch, but rather a prerequisite for the fine-grained alignment to yield consistent gains. Without temporal and structural constraints, fine-grained matching itself introduces noise due to unordered frame correspondence, thereby weakening the model’s discriminative capability.

#### 4.2.4. Computational Cost

We also evaluate the computational overhead introduced by SASA-CLIP’s specialized components: the fine-grained branch, GPGA module, adaptive fusion, and the LLM descriptor bank. Table 10 benchmarks our method against three standard CLIP-based models (X-CLIP, OST, and BIKE). All models were tested under identical configurations (ViT-B/32, T=8 frames, 224×224 resolution, batch size 1) on a single RTX 4090D GPU.

Admittedly, improving video action recognition accuracy comes with a computational cost. Table 10 shows that SASA-CLIP’s structural complexity brings the FLOPs to 41.03 G and peak inference memory to 810 MB, which are slightly higher than those of the baseline models. The larger total parameter count of SASA-CLIP (203.85 M) mainly comes from retaining the full CLIP encoders. However, the trainable parameter count (140.29 M) remains reasonably constrained—heavier than X-CLIP (131.50 M) but still avoiding the full fine-tuning cost required by OST (151.28 M). The main additional cost is inference latency (18.89 ms per video), which arises from the fine-grained frame-level matching and the GPGA module. On 4 × RTX 4090D, SASA-CLIP takes about 38 min per epoch on Kinetics-400, close to the 34 min per epoch of X-CLIP, suggesting that the added modules introduce only a small training overhead. An efficient and deployment-oriented model design is an important consideration for real-world visual sensing applications [32], which motivates the computational analysis reported in this section.

### 4.3. Supervised Experiments

As shown in Table 11, we evaluate our method on the large-scale Kinetics-400 dataset and compare it with recent CLIP-based video recognition methods, including ActionCLIP, Text4Vis, OST, X-CLIP, and BIKE. All methods adopt a unified input setting of T=8 frames at 224×224 resolution, and are evaluated under both ViT-B/32 and ViT-B/16 backbones.

Under the ViT-B/32 backbone, our method achieves a Top-1 accuracy of 81.37%, an improvement of 0.97% over the X-CLIP baseline (80.40%), and outperforms contemporary methods including ActionCLIP (78.36%), Text4Vis (78.50%), and OST (78.70%). Compared with BIKE (81.40%), our method performs comparably (−0.03%), reaching the same accuracy level. Considering that BIKE achieves its performance gain through an additional bidirectional cross-modal knowledge distillation mechanism, whereas our improvement arises entirely from the cooperative design of multi-granularity semantic prototypes on the textual side and GPGA-based temporal alignment, this result suggests that explicit semantic decomposition combined with structure-aware temporal alignment can achieve competitive performance without relying on complex cross-modal distillation mechanisms.

Under the ViT-B/16 backbone, our method achieves 84.23% Top-1 accuracy, surpassing X-CLIP (83.80%) by 0.43% and BIKE (84.00%) by 0.23%, and outperforming ActionCLIP (81.09%), Text4Vis (81.40%), and OST (82.00%). Notably, the advantage of our method becomes more pronounced as the backbone capacity scales from ViT-B/32 to ViT-B/16: while our method performs comparably to BIKE under ViT-B/32, it surpasses BIKE by 0.23% under ViT-B/16. This indicates that the proposed fine-grained semantic alignment mechanism can benefit more effectively from stronger visual representations, thereby further improving discriminative capability in large-scale action recognition.

Overall, our method achieves competitive performance with recent CLIP-based methods under the Kinetics-400 fully supervised setting. Importantly, this improvement arises entirely from a more thorough exploitation of the spatio-temporal semantic structure already embedded in CLIP’s pre-trained knowledge, without introducing additional cross-modal distillation or large-scale vision–language alignment.

We further evaluate our method on the HMDB-51 and UCF-101 datasets, with results shown in Table 12. The experiments in this section are conducted by the authors. All methods adopt ViT-B/16 as the backbone and are trained for 30 epochs under the same experimental setting to ensure fair comparison. Since X-CLIP and BIKE do not report fully supervised results on HMDB-51 and UCF-101 in their original papers, we reproduce these baselines using their official open-source code under our experimental setting.

On the HMDB-51 dataset, our method achieves a Top-1 accuracy of 74.00% under T=16, an improvement of 3.25% over the X-CLIP baseline (70.75%) and surpassing BIKE (73.31%) by 0.69%. Our method helps most on fine-grained actions, and that makes sense, because their phase structures are complex and the distinguishing cues lie in small differences between phases.

On the UCF-101 dataset, our method also delivers strong results. Under T=16, it reaches a Top-1 accuracy of 96.81%, an improvement of 2.61% over X-CLIP (94.20%), and surpasses BIKE (96.63%) by 0.18%. Under the more challenging T=8 configuration, our method (96.65%) further widens its margin over BIKE (96.15%) to 0.50%. These results suggest that the proposed fine-grained alignment strategy remains effective even when fewer input frames are available.

On both HMDB-51 and UCF-101, then, SASA-CLIP comes out ahead of the X-CLIP baseline and of BIKE alike. This validates that the multi-granularity semantic prototypes combined with the GPGA-based temporal alignment mechanism generalize well across action recognition datasets of varying scales and characteristics.

### 4.4. Zero-Shot Experiments

As shown in Table 13, we conducted zero-shot experiments on the classic zero-shot datasets HMDB-51 and UCF-101 to evaluate the zero-shot performance of our model. The experimental results demonstrate consistent improvements over the baseline model. For the experiments in Section 4.4, we first pre-trained the model on the Kinetics-400 dataset using four NVIDIA RTX 4090D GPUs, adopting the ViT-B/16 backbone with a 32-frame input configuration. After completing the pre-training on the Kinetics-400 dataset, zero-shot evaluations were performed on HMDB-51 and UCF-101.

As shown in Table 13, SASA-CLIP achieves a Top-1 accuracy of 75.1% on the UCF-101 dataset, yielding a 3.1% improvement over the baseline. On the HMDB-51 dataset, it achieves a Top-1 accuracy of 48.9%, an improvement of 4.3% over the baseline. These results indicate that the proposed SASA-CLIP method is not only effective under fully supervised training but also exhibits promising generalization capability in the zero-shot setting.

It is worth noting that OST [7] achieves a Top-1 accuracy of 79.7% on the UCF-101 dataset and 55.9% on the HMDB-51 dataset, both of which outperform our method. This difference stems from the different design orientations of the two methods, as analyzed below. OST solves the frame-descriptor matching via the Sinkhorn global optimal transport algorithm, which does not assume a temporal structure and estimates the correspondence between frames and descriptors for each sample. This adaptability accounts for its higher zero-shot results. In contrast, the temporal centers $\mu_{j}$ of SASA-CLIP are fixed by the phase indices of the descriptors (Equation (6)), with only the fusion coefficient α learnable. During supervised training, these fixed centers work together with sufficient samples from the seen categories and guide the encoder to learn discriminative temporal representations. Under zero-shot transfer, however, the temporal structure of an unseen category does not necessarily follow the assumed start-middle-end structure, and the prior centers cannot be adjusted to it. A constraint that is beneficial under supervision therefore becomes a mismatched constraint on unseen categories. This reflects a design trade-off. The deterministic temporal prior improves discrimination under supervision, but at the cost of adaptation to unseen categories. The parameter-free matching of OST adapts more readily to unseen categories, while providing weaker temporal discrimination under supervision. Under the fully supervised setting on Kinetics-400 with ViT-B/32, SASA-CLIP outperforms OST by 2.67% (Table 11). Enhancing zero-shot generalization while retaining the temporal structural constraint, for example by introducing learnable or dynamically adjusted prior parameters, remains a direction for future work.

## 5. Conclusions

In this paper, we propose SASA-CLIP, a structure-aware and semantically adaptive framework for fine-grained vision–language alignment. It aims to address the insufficient discriminability of existing CLIP-based video action recognition methods for fine-grained actions. Through a dual-branch architecture, SASA-CLIP shifts cross-modal alignment from the global pooled feature-descriptor level to the frame-descriptor level. The coarse-grained branch preserves global contextual features, while the fine-grained branch utilizes spatio-temporal descriptors generated by an LLM to achieve frame-level semantic matching. Concurrently, to overcome the temporal disorder in frame-wise alignment, we propose the Gaussian-Prior Guided Attention (GPGA) module, which explicitly encodes the inductive bias of local temporal continuity into the attention weights and guides the ordered alignment between temporal descriptors and their corresponding video phases. Experiments on Kinetics-400, HMDB-51, and UCF-101 show consistent gains under both fully supervised and zero-shot settings. Beyond benchmark evaluation, SASA-CLIP offers a practical approach for fine-grained action understanding in real-world visual sensing applications such as intelligent surveillance and wearable activity monitoring.

Nevertheless, our method still has certain limitations that are worth exploring in future work. First, the performance of the model depends on the quality of the LLM-generated descriptors, as reflected in the residual error analysis in Section 4.2.1. Future work could introduce an adaptive filtering mechanism based on matching scores to filter out low-quality descriptors. Second, GPGA fixes the temporal centers $\mu_{j}$ and shares a single standard deviation σ across all of them. This rigidity is less suited to action categories whose temporal structure varies widely. Future research could explore learnable parameters or strategies that dynamically adjust parameters based on inter-frame feature similarity. Third, there remains a performance gap in the zero-shot setting between our method and optimal transport-based methods such as OST. This likely reflects a potential trade-off between the discriminative gains introduced by temporal structural priors during supervised training and the flexibility required for zero-shot generalization. Enhancing zero-shot generalization while retaining temporal structural constraints remains an open direction for future work. Following common practice for this dataset, the Kinetics-400 results are reported from single training runs, as repeated full training is computationally expensive. The consistency of the gains across backbones, frame numbers, and datasets supports the reliability of the improvement.

## Figures and Tables

**Table 1:** Comparison of representative video action recognition methods.

Method	Datasets	Methodology	Feature Extraction	Performance Evaluation
I3D [11]	Kinetics-400	Two-stream inflated 3D convolution	Inflated 3D ConvNet	Closed-set supervised; 71.10% on K400
TimeSformer [16]	K400, SSv2	Divided space-time self-attention	ViT (Transformer)	Closed-set supervised; 78.00% on K400
ActionCLIP [4]	Kinetics-400	Pre-train, prompt, fine-tune	CLIP ViT-B/16	Supervised & zero-shot; 81.09% on K400
X-CLIP [5]	K400, HMDB-51, UCF-101	Cross-frame communication	CLIP ViT-B/16	Supervised & zero-shot; 83.80% on K400
ViFi-CLIP [6]	K400, HMDB-51, UCF-101	Full fine-tuning + frame averaging	CLIP ViT-B/16	Supervised & zero-shot; 83.90% on K400
OST [7]	K400, UCF-101, HMDB-51	LLM descriptors + Sinkhorn optimal transport	CLIP ViT-B/16	Supervised & zero-shot; 82.00% on K400
BIKE [19]	K400, HMDB-51, UCF-101	Bidirectional cross-modal knowledge distillation	CLIP ViT-B/16	Supervised & zero-shot; 84.00% on K400

**Table 2:** Empirical analysis of CLIP’s temporal agnosticism on the Kinetics-400 validation set.

Method	Start	Middle	End
Expected μ	0.00	0.50	1.00
Empirical μ (validation set)	0.49	0.49	0.49

**Table 3:** Detailed information on datasets.

Dataset	Categories	Training Set	Test Set	Total	Split	Sources
HMDB-51 [30]	51	3570	1530	6849	3	Movies and web videos
UCF-101 [31]	101	9537	3783	13,320	3	YouTube
Kinetics-400 [11]	400	∼240k	∼20k	∼260k	1	YouTube

**Table 4:** Detailed settings of model training hyperparameters.

Setting	Value
*Training Hyperparameters*	
GPUs	4 × NVIDIA RTX 4090D
Batch size	256 (fully supervised), 64 (zero-shot)
Training epochs	30
Optimizer	AdamW, β=(0.9,0.98), $\epsilon =10^{-8}$
Weight decay	$1\times 10^{-3}$
LR schedule	Cosine annealing
Warmup epochs	5
LR (CLIP backbone)	$8\times 10^{-6}$ (K400), $2\times 10^{-6}$ (HMDB-51, UCF-101)
LR (new modules, 10×)	$8\times 10^{-5}$ (K400), $2\times 10^{-5}$ (HMDB-51, UCF-101)
σ (Gaussian prior)	0.25
$\tau_{att}$ (attention temperature)	0.2
$\tau_{con}$ (contrastive temperature)	0.07 (initialized, learnable)
*Data Augmentation*	
Training resize	RandomSizedCrop
Training crop size	224
Random horizontal flip	probability 0.5
Color jitter	probability 0.8
Random grayscale	probability 0.2
Mixup α	0.8
CutMix α	1.0
Switch probability	0.5
Label smoothing	0.1

**Table 5:** Performance comparison of different branch configurations on the Kinetics-400 dataset.

Branch Configuration	Backbone	Input	Top-1 (%)	Top-5 (%)
X-CLIP [5]	ViT-B/32	8 × 224 × 224	80.40	95.00
		16 × 224 × 224	81.10	95.50
	ViT-B/16	8 × 224 × 224	83.80	95.70
		16 × 224 × 224	84.70	96.80
SASA-CLIP (Ours)	ViT-B/32	8 × 224 × 224	81.37	95.35
		16 × 224 × 224	81.70	95.67
	ViT-B/16	8 × 224 × 224	84.23	95.78
		16 × 224 × 224	85.60	97.04

**Table 6:** Contribution of the coarse-grained and fine-grained branches on the Kinetics-400 dataset.

Branch Configuration	Fusion (λ)	Backbone	*T*	Top-1 (%)	Top-5 (%)
X-CLIP [5] (baseline)	–	ViT-B/32	8	80.40	95.00
SASA-CLIP (coarse only)	λ=0			80.53	95.09
SASA-CLIP (fine only)	λ=1			79.82	94.56
SASA-CLIP (Full)	adaptive			81.37	95.35

**Table 7:** Ablation on descriptor types and fusion strategy on Kinetics-400. ✓ indicates that the corresponding component is enabled and × indicates that it is disabled.

Configuration	Spatial Desc	Temporal Desc	GPGA	Fusion	Top-1 (%)	Top-5 (%)
Spatial only	✓	×	×	Fixed	80.24	94.97
Temporal only	×	✓	✓	Fixed	80.71	95.18
Spatial + Temporal (Full)	✓	✓	✓	Fixed	81.06	95.23
Spatial + Temporal (Full)	✓	✓	✓	Adaptive	81.37	95.35

**Table 8:** Ablation on the number of spatial descriptors on Kinetics-400.

M_s_ (Spatial)	M_t_ (Temporal)	Backbone	T	Top-1 (%)	Top-5 (%)
1	3	ViT-B/32	8	81.14	95.28
3	3			81.37	95.35
5	3			81.21	95.26
7	3			81.07	95.12

**Table 9:** Ablation study of the GPGA module on the Kinetics-400 dataset. All experiments use ViT-B/32 as the backbone. ✓ indicates that the corresponding component is enabled and × indicates that it is disabled.

Configuration	Coarse-Grained Branch	Fine-Grained Branch	GPGA	Input	Top-1 (%)/Top-5 (%)
X-CLIP (Baseline)	✓	×	×	8 × 224 × 224	80.40 / 95.00
				16 × 224 × 224	81.10 / 95.50
SASA-CLIP w/o GPGA	✓	✓	×	8 × 224 × 224	80.81 / 94.67
				16 × 224 × 224	81.23 / 95.10
SASA-CLIP (Full)	✓	✓	✓	8 × 224 × 224	81.37 / 95.35
				16 × 224 × 224	81.70 / 95.67

**Table 10:** Computational cost comparison on Kinetics-400.

Method	Total (M)	Trainable (M)	FLOPs (G)	Latency (ms)	Memory (MB)
X-CLIP [5]	196.60	131.50	39.00	12.01 ± 0.48	781
OST [7]	151.28	151.28	35.32	06.75 ± 0.22	611
BIKE [19]	171.28	107.85	38.78	13.53 ± 0.15	786
SASA-CLIP	203.85	140.29	41.03	18.89 ± 0.71	810

**Table 11:** Performance comparison between SASA-CLIP and other alternative methods on the Kinetics-400 dataset.

Methods	Input	Backbone	K400 Top-1 (%)
ActionCLIP [4]	8 × 224 × 224	ViT-B/32	78.36
		ViT-B/16	81.09
Text4Vis [33]	8 × 224 × 224	ViT-B/32	78.50
		ViT-B/16	81.40
OST [7]	8 × 224 × 224	ViT-B/32	78.70
		ViT-B/16	82.00
X-CLIP [5]	8 × 224 × 224	ViT-B/32	80.40
		ViT-B/16	83.80
BIKE [19]	8 × 224 × 224	ViT-B/32	81.40
		ViT-B/16	84.00
SASA-CLIP (Ours)	8 × 224 × 224	ViT-B/32	81.37
		ViT-B/16	84.23

**Table 12:** Performance comparison between SASA-CLIP and reproduced baselines (X-CLIP and BIKE) on the HMDB-51 and UCF-101 datasets. All methods use ViT-B/16 as the backbone, trained for 30 epochs in our experimental environment.

Methods	Backbone	T	Epoch	HMDB-51 (%)	UCF-101 (%)
BIKE	ViT-B/16	8	30	72.22	96.15
		16		73.31	96.63
X-CLIP		8		70.22	94.10
		16		70.75	94.20
Ours		8		72.68	96.65
		16		74.00	96.81

**Table 13:** Zero-shot performance comparison between SASA-CLIP and other alternative methods on the UCF-101 dataset and HMDB-51.

Methods	Backbone	T	UCF-101 Top-1 (%)	HMDB-51 Top-1 (%)
ActionCLIP [4]	ViT-B/16	32	58.3	40.8
ViFi-CLIP [6]		32	76.8	51.3
Vita-CLIP [34]		32	75.0	48.6
X-CLIP [5]		32	72.0	44.6
OST [7]		32	79.7	55.9
SASA-CLIP (ours)		32	75.1	48.9

## Data Availability

This study uses three publicly available benchmark datasets. Kinetics-400 is available at https://github.com/cvdfoundation/kinetics-dataset (accessed on 1 June 2026); HMDB-51 is available at https://serre-lab.clps.brown.edu/resource/hmdb-a-large-humanmotion-database/ (accessed on 1 June 2026); and UCF-101 is available at https://www.crcv.ucf.edu/data/UCF101.php (accessed on 1 June 2026). No new data were created in this study. The code and trained models are available from the corresponding author upon reasonable request.

## Supplementary Materials

The prompt templates used for descriptor generation, together with the complete set of spatial and temporal descriptors for all action categories, are publicly available at https://github.com/siwenbao/SASA-CLIP (accessed on 1 June 2026).
